# Japanese health and safety information for overseas visitors: a randomized controlled trial

**DOI:** 10.1186/s12889-023-16117-5

**Published:** 2023-06-21

**Authors:** Mariko Nishikawa, Masaaki Yamanaka, Akira Shibanuma, Junko Kiriya, Masamine Jimba

**Affiliations:** 1 Department of Global Health and Nursing, Graduate School of Nursing, The University of Human Environments, Nagoya, Japan; 2 3-220, Ebata Cho, Obu City, 474-0035 Aichi Japan; 3 Department of Maritime Science and Technology, Japan Coast Guard Academy, Kure, Japan; 4 Department of Community and Global Health, Graduate School of Medicine, The University of Tokyo, Tokyo, Japan

**Keywords:** CSQ-8, Health education, Game, Japan, Overseas visitors

## Abstract

**Introduction:**

International travel to Japan increased steadily until the coronavirus disease 2019 (COVID-19) outbreak. Although international travel was curtailed worldwide due to the pandemic, the number of overseas visitors to Japan should increase again after the restrictions are lifted. We assessed the effect of a five-minute digital game on the knowledge of health information and the level of satisfaction with educational health resources of overseas visitors to Japan*.*

**Methods:**

We conducted a randomized controlled trial among 1062 previous and potential visitors to Japan utilizing an internet portal. We recruited previous and potential visitors to Japan from the internet portal sites of the UK, the US and Australia. We randomly allocated participants to two groups: an intervention group that played an animated game and a control group that viewed an online animation. All participants answered a self-administered questionnaire online from March 16 to 19, 2021. We assessed visitors’ levels of health knowledge and satisfaction using the CSQ-8. We analyzed the data with a t test and the difference in differences test. Our RCT followed the SPIRIT guidelines.

**Results:**

Of the 1062 previous and potential visitors recruited via the three countries’ internet portals (354 from each country), some had visited Japan previously (174 in the intervention group, 220 in the control group), while some were potential visitors to Japan (357 in the intervention group, 311 in the control group). Some had gathered health and safety information about Japan prior to this study (180 in the intervention group, 211 in the control group). Both groups improved their health information levels after the intervention. The level of satisfaction with health information in Japan was significantly increased in the intervention group (average difference of 4.5 points) compared to that in the control group (average difference of 3.9 points) (*p* < 0.05). Both groups’ mean CSQ-8 scores increased significantly after the intervention (*p* < 0.001): from 23 to 28 in the intervention group and from 23 to 24 in the control group.

**Conclusions:**

Our study introduced unique educational strategies using an online game to provide health and safety information to previous and potential visitors to Japan. The online game was a more effective way to increase satisfaction than the online animation about health information.

This study was registered in the UMIN-CTR (University Hospital Medical Information Network Center Clinical Trials Registry) as Version 1, and the trial registration data are available as UMIN000042483, 17/11/2020.

**Trial registration:**

Trials UMIN-CTR (University Hospital Medical Information Network Center Clinical Trials Registry), UMIN000042483 (Japanese health and safety information for overseas visitors: A randomized controlled trial), 17/11/2020.

**Supplementary Information:**

The online version contains supplementary material available at 10.1186/s12889-023-16117-5.

## Introduction

Although international travel was greatly reduced for approximately three years due to the coronavirus disease 2019 (COVID-19) pandemic in early 2020, it is expected to rebound to pre pandemic levels in a globalized and interconnected economy [1]. The number of overseas visitors to Japan has steadily increased over the last decade from 8.6 million in 2010 to 31.8 million in 2019 [2]. The rapid increase in international visitors could lead to an increase in the number of those who fall sick or are injured during their travel and must visit a health facility in Japan.

International visitors can access information about disease prevention and its health care system if they need to receive any health care service [3]. A study conducted in 2003 examined how overseas visitors obtained information before traveling to low- and middle-income countries [3]. Although health risks are not confined to those specific countries, visitors are considered susceptible to infectious diseases and are required to be vaccinated before traveling [4]. Even when visiting a high-income country, it is important to obtain health information about the country.

Our previousstudy found that only 18% (45 of 265) of overseas visitors had access to information about the Japanese health care system before coming to Japan [5]. In our earlier study among 747 people from 53 countries at international airports, we surveyed the concerns of overseas visitors should they fall ill. The top three concerns were language, insurance, and informed consent. A total of 6.3% of respondents needed to visit a hospital for illness or injury during their visit to Japan. The number of respondents who did not hold medical insurance was 22.6% (168 of 747) [5]. As this situation continues to evolve, Japan’s unique language, culture, and health system will become strained in dealing with an unprecedented number of international visitors. To ameliorate this situation, it is important to develop health information guidelines that address the concerns of international visitors and provide necessary health information about the country being visited.

There are two main concerns related to the low level of health information accessed by overseas visitors to Japan. First, the majority of overseas visitors to Japan are young adults (a median age of 30–39 years) [5]. Young adults travel more extensively and participate in more rigorous activities, such as mountain climbing and skiing, compared to older age groups. They are at higher risk of injury, even though they consider themselves less likely to be injured while traveling [6]. Providing health information would be beneficial to both visitors and health care facilities in host countries, but we have not found any studies that address this.

Second, the usefulness of pretravel health information depends upon the dissemination method and content of the information [7, 8]. Overseas visitors usually receive health information through travel books, websites, and pamphlets or by visiting clinics in their home countries [7, 9, 10]. Presently, the public health organizations of numerous countries provide health and safety information. Health information is provided at different places but may be insufficient for all visitors. Additionally, infectious diseases and immunizations are the main focus of the information for countries visited [11–13]. Thus, it is important to disseminate information more efficiently to international visitors and to test its effectiveness.

Developments in digital technology have resulted in changes in the provision of information in several languages and in various formats [14]. This is suitable for most visitors, including those at risk of injury when visiting foreign countries. A digital game can be a desirable way to visually present culturally important information [15–17]. In a previous study, a digital game for general surgery medical residents in classrooms demonstrated a significant increase in short- and long-term medical knowledge and elicited high levels of student satisfaction [18]. Another study that involved instructing nursing students found that an educational game was more enjoyable than conventional formats and was accepted by learners as a satisfying teaching technique [19].

We explored the effectiveness of a digital animation and digital game through intervention studies in stages. First, we developed a video animation of health care services based on our preliminary research [5]. By comparing this animated video with an existing guidebook, we found that the intervention was effective in reducing concerns about staying healthy in Japan [20]. Then, we attempted to test the effectiveness of the animation with a five-minute game in the form of a quiz. This was expected to increase knowledge about having a safe and healthy stay in Japan.

We tested a short-term intervention because a study showed that short educational videos can improve clinical skill acquisition for medical students [21]. The game was five minutes long for three reasons. First, to explain the objective, encourage participation, and complete the game. Second, a shorter duration allowed participants to focus on their responses. Third, the participants were able to remember the information provided during the intervention until the postintervention survey. The objective of this study was to examine the efficacy of an animated online game titled *Sa-Chan game* on knowledge and satisfaction and the motivation to follow instructions regarding health information among previous and potential visitors to Japan.

## Methods

### Study design and procedures

We conducted a randomized controlled trial to determine the efficacy of a five-minute online animated game on improving knowledge among previous and potential visitors to Japan.

**Fig. 1:**
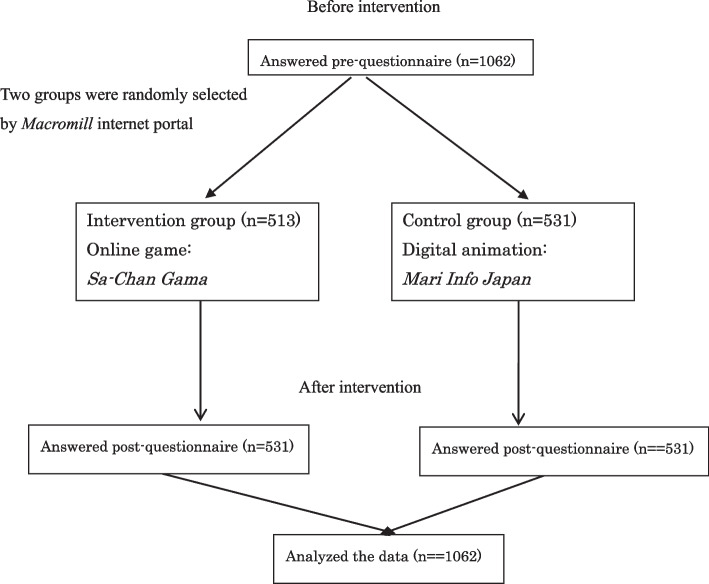
Schematic design of study

We also examined their satisfaction with the information provided and their willingness to follow the information. The participants completed an online survey. They responded to a questionnaire on the above outcomes prior to and after receiving either of the interventions. We then assessed changes in the outcomes (Fig. 1).

## Participants

### Sample size

The sample size was calculated as 1,000 based on a previous study on the provision of health care information among international tourists to Japan [20], which showed a standardized mean difference in the level of anxiety of 0.178 (standard deviation = 1.0) under a 95% significance level and 80% power.

### Eligibility

Individuals who were previous or potential visitors to Japan from the United Kingdom (UK), the United States (US), and Australia were recruited through a website. These individuals consented to participate in our study after reading our objectives and prior to answering the questionnaire. The survey was written in English to avoid biases regarding interpretation. This study was conducted in English to avoid multilingual interpretations of the information provided and follow-up questions. When recruiting participants, we ensured that they had sufficient English ability to understand the contents of the intervention, quiz, and questionnaire. Their English level was self-reported at the time of registration. To ensure the validity of the responses to our questionnaire, the survey targeted those who were 18 years or older, could understand English, and had visited or were planning to visit Japan.

### Enrollment procedure

The process for participation in this study was as follows. First, the study content, conditions for participation, and request for participation were sent via e-mail; second, willingness to participate was confirmed by e-mail replies; third, those who confirmed their participation were randomly assigned by the computer to either the intervention or the control group in a prospective manner and stratified by country, sex, and age. Ineligible individuals were excluded from participation at the beginning of this study.

The respondents registered online before participating in this study. The participants accessed the questionnaire site by accepting an e-mail invitation. The online system objectively determined the eligibility of those who accessed the website based on their background information provided at registration. Only those who met the inclusion criteria of this study could complete our questionnaire; the criteria were explained, and the questionnaire was made available after the participants provided their consent.

The online system was set up to equally and randomly allocate participants (1:1) to either the intervention group or the control group stratified by sex (male vs. female) and age (18–29, 30–39, 40–49, 50–59, over 60). After a participant answered all questions, the system ensured that the participant could not repeat the program. The online system was closed when the required sample size was reached. We asked the participants to respond to the questions based on their current knowledge and not to search for answers elsewhere. The individuals received a gift certificate for one British pound sterling, one US dollar or one Australian dollar from the *Macromill* Company upon completing the questionnaire from March 16 to 19, 2021.

## Interventions

### Intervention group

The intervention group participated in a five-minute game titled *Sa-Chan game* in English (Table 1) [22]. We administered the animated game through a website. The game was designed as a quiz that aimed to supply information on disease prevention and safety in Japan for overseas visitors. We created the game based on the results of a previous study on overseas visitors’ concerns about health information in Japan [23]. The game started with East Asian music and contained 11 topics. After the participants answered each question, the screen showed the correct answers and explanations, and the participants received a total score after all questions were answered.

**Table 1:**
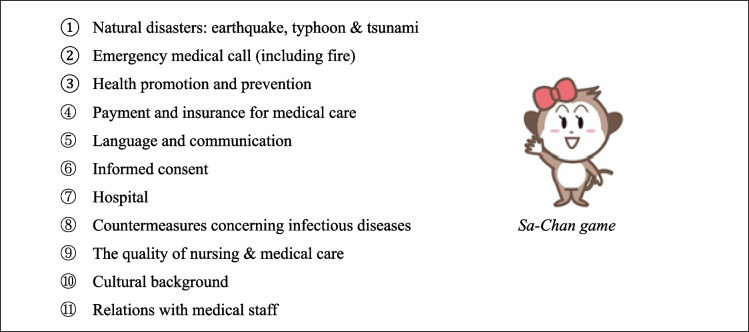
Intervention group. *Sa-Chan game*: Contents of Japanese health & safety game for overseas visitors

### Control group

The control group watched a four-minute cartoon in English named *Mari Info Japan* (Table 2) through a website [22]. The purpose was to deliver information about the health care system in Japan to overseas visitors [23]. The cartoon covered 11 topics.

**Table 2:**
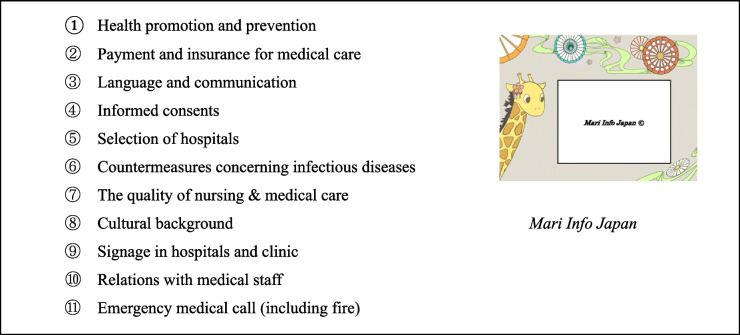
Control group. *Mari Info Japan:* Contents of Japanese health & safety information for overseas visitors

## Outcomes

The primary outcome was whether visitors’ knowledge of the Japanese health system improved through the *Sa-Chan game* or *Mari Info Japan*. We asked all participants in both groups to complete a short questionnaire to assess their knowledge of the Japanese health system before and after the interventions. Since the results of our analyses showed that there was little impact of participants’ previous visits to Japan on their outcome scores, we considered the bias to be small and conducted the analyses together in this study. If a participant understood the information of each of the 11 topics (15 items), he or she answered “yes”.

The secondary outcome of this study was the average CSQ-8 (8-item Client Satisfaction Questionnaire) [24] score between the visitors who had participated in the *Sa-Chan game* and those who watched *Mari Info Japan* immediately after the interventions. We evaluated the outcome by providing a self-administered questionnaire, the CSQ-8, which has been widely used for health education [24] and was indicated to be reliable and valid in earlier research [25]. The CSQ-8 uses a four-point Likert scale, and in this study, it was used to measure the respondent’s level of satisfaction with health promotion and safety in Japan. The score ranged from 8 to 32. A higher score indicated better satisfaction.

The third outcome of this study was participants’ willingness to follow the provided information immediately after the interventions. We asked the question, “Are you likely to follow the information yourself”, and the visitors answered using a four-point Likert scale to indicate whether they were willing to follow the Japanese health-related information. We referred to the information-motivation-behavioral (IMB) skills model to ask this question [26]. This model has been applied in a number of risk reduction behavior studies [27]. The intervention and completion of the questionnaire took approximately 10 min.

## Other information

Furthermore, we asked about the background characteristics of the participants: country, sex, age, education level, whether they had received health information about Japan before this study, and the source of the information. We checked whether the distributions of background characteristics were similar between the two groups.We performed a pilot test with 13 students and professors at a college in New York to verify whether they understood the content of the questionnaire on 12 August 2018.

## Blinding

Both the participants and the researchers who collected the data were blinded to the allocation of participants. Two researchers (MN and AS) analyzed the data independently to minimize bias.

## Data analysis

The difference in the primary outcome, the participants’ knowledge of the Japanese health system gained after playing the *Sa-Chan game* and *Mari Info Japan*, was tested by a t test.

For the secondary outcome, we examined the change in the mean CSQ-8 scores recorded pre- and post-intervention between the groups with a t test. To evaluate the pre- and postintervention scores between groups, the difference in differences test was used. We used Cronbach’s alpha to test the reliability of the questionnaire.

For the third outcome regarding willingness, we compared the pre- and postintervention and between-group behavioral score changes with the chi-square test. All data analyses were performed using the JMP statistical package (version 14.0, SAS Campus Drive, Cary, NC 27513, USA).

## Results

We recruited 1062 international prior or potential visitors to Japan who agreed to participate in this study. We analyzed the data of 531 participants in the intervention group and 531 participants in the control group.

Table 3 shows a summary of the basic characteristics of both groups. The median age was 40–49 years, with 528 females (49.7%) and 534 males (50.3%). The groups were balanced with respect to baseline characteristics: age, sex, country, education level, and plans to visit Japan after COVID-19. However, three variables had greater values in the control group: previous visits to Japan (*p* < 0.01), frequency of visiting Japan (*p* < 0.001), and previous health and safety information received (*p* < 0.001). Over one-third of the participants had visited Japan once or more often in both groups. Among the participants, 349 (65.7% of the total) in the intervention group and 320 (60.3% of the total) in the control group had not obtained Japanese health information before this study. Their sources of information were the internet, health facilities, travel guidebooks, brochures, and others for both groups. A total of 503 (94.7%) participants in each group had plans to visit Japan after COVID-19.

**Table 3:** Demographic profile of participants

						*N* = 1062
Variable		Intervention *n* = 531		Control *n* = 531		
		n	(%)	n	(%)	*p*
Age	18–29	108	(20.3)	108	(20.3)	
	30–39	108	(20.3)	108	(20.3)	
	40–49	108	(20.3)	108	(20.3)	0.999
	50–59	105	(19.8)	105	(19.8)	
	Over 60	102	(19.3)	102	(19.3)	
Gender	Female	264	(49.7)	264	(49.7)	0.999
	Male	267	(50.3)	267	(50.3)	
Country	USA	177	(33.3)	177	(33.3)	0.999
	UK	177	(33.3)	177	(33.3)	
	AU	177	(33.3)	177	(33.3)	
Education	University and more	280	(52.7)	295	(55.6)	0.622
	Junior college	146	(27.5)	134	(25.2)	
	High school	105	(19.8)	102	(19.2)	
Visited Japan	Yes	174	(32.8)	220	(41.1)	0.004**
	No	357	(67.2)	311	(58.6)	
Frequency to visit Japan	Once	113	(64.9)	143	(65.0)	0.001***
	More than once	61	(35.1)	77	(35.0)	
Got health and safety information	Yes	180	(34.3)	211	(39.7)	0.001***
	No	349	(65.7)	320	(60.3)	
Source of the information^a^	Internet	166		196		
(Not only Japan)	Health facility	68		76		
	Travel guidebook	52		63		
	Brochure	45		52		
	Others	9		10		
Plan to visit Japan after COVID-19	Yes	503	(94.7)	503	(94.7)	0.999
	No	28	(5.3)	28	(5.3)	

^a^Multiple answers

X2 **p < 0.01, ***p < 0.001

Table 4 shows the participants’ understanding of how to follow information related to the 11 topics (15 items) in Japan before and after the interventions. Both groups understood the health information for Japan after the intervention. The three items with the highest scores for understanding in the intervention group included the medical system, the quality of nursing care, and dealing with medical staff. The three items with the highest scores for understanding in the control group were choosing a hospital, the medical system, and dealing with medical staff.

**Table 4:** Understanding health information topics compared before-after interventions for both groups

								*N* = 1062
	Intervention = 531				Control = 531			
11 topics (15 items)	before	after	after-before	*p*	before	after	After-before	*p-*value
Medical system	244	479	235	0.001	260	492	232	0.001
Paying medical expenses	249	451	202	0.001	251	488	237	0.020
Quality of nursing care	275	496	221	0.048	287	491	204	0.001
Quality of the medicine	332	469	137	0.001	351	462	111	0.001
Dealing with medical staff	267	484	217	0.043	274	496	222	0.001
Choosing a hospital	232	441	209	0.001	238	478	240	0.049
Language communication	285	470	185	0.001	308	484	176	0.004
Emergency care	301	482	181	0.001	286	485	199	0.001
Directions in a hospital	240	420	180	0.001	255	462	207	0.001
Picking up an infection	281	454	173	0.048	279	467	188	0.001
Informed consent	327	488	161	0.001	318	497	179	0.003
Lifestyle differences	406	486	80	0.001	407	481	74	0.001
Eye contact	344	501	157	0.001	359	496	137	0.006
Protection of privacy	328	474	146	0.001	342	452	110	0.001
Health promotion and safety	362	487	125	0.001	354	484	130	0.001

Each number = understanding (Yes) t test

In the intervention group, the participants’ backgrounds regarding gathering information before this study and the number of times they visited Japan were correlated with understanding information topics. In the control group, the participants’ background regarding gathering information before this study was correlated with understanding information topics through the chi-square test.

Table 5 shows that the CSQ-8 scores improved in both groups after the interventions. A statistically significant increase in satisfaction was observed in the intervention group (mean score changed from 23 to 28, *p* < 0.001) and the control group (mean score changed from 23 to 27, *p* < 0.001).

**Table 5:** ^a^CSQ-8 outcomes for before vs. after test in each group

	Before					After						*N* = 1062
Items	^b^M	^c^(SD)	Standard error	95% confidence interval		^b^M	^c^(SD)	Standard error	95% confidence interval		Average difference	*p*-value
				Lower	Upper				Lower	Upper		
Intervention group = 531	23	(4.9)	0.214	22.902	23.742	28	(3.9)	0.169	27.523	28.187	4.533	0.001
(*Sa-Chan game*)												
Control group = 531	23	(5.0)	0.219	22.841	23.701	27	(4.5)	0.197	26.790	27.563	3.906	0.001
(*Mari Info Japan*)												

^a^*CSQ-8* 8-item Client Satisfaction Questionnaire, ^b^*M* Mean, ^c^*SD* Standard Deviation t-test

Table 6 shows the comparison of the two groups after the interventions through difference in difference analyses. The CSQ-8 scores increased more in the intervention group than in the control group (mean difference: 4.5 in the intervention group and 3.9 in the control group, *p* < 0.041). We found that the CSQ-8 scores were not significantly different between the countries (the US, the UK and Australia).

**Table 6:** ^a^CSQ-8 outcomes for intervention vs. control group

	Intervention = 531				Control = 531				*N* = 1062
Items	Average difference	Standard error	95% confidence interval		Average difference	Standard error	95% confidence interval		*p*-value
			Lower	Upper			Lower	Upper	
^a^CSQ-8	4.5	0.216	4.108	4.958	3.9	0.216	3.482	4.330	0.041

^a^*CSQ-8* 8-item Client Satisfaction Questionnaire

Difference in difference analysis

Table 7 shows the results of the multiple regression analysis. In the intervention group, the CSQ-8 scores were higher among women, those aged 60–69 years, those who had never visited Japan, and those who had not previously received health information. In the control group, the CSQ-8 scores were higher among women, those who had never visited Japan, and those who had not previously received health information. The adjusted R^2^ was 0.129. In this study, the CSQ-8 was confirmed to have high reliability, with a Cronbach’s alpha internal consistency reliability coefficient of 0.936 after the interventions.

**Table 7:** Correlation and multiple regression analysis of various factors in relation to ^a^CSQ-8 scores

			(*N* = 1062)	
Items	*R*^*2*^	*AdjustR*^*2*^	^*b*^*SE*	*p-*value
Intervention: *Sa-Chan game* (*n* = 531)	0.2048	0.1911		
Gender			0.0140	0.014
Age (18-20 s: 1, 30 s: 2, 40 s: 3, 50 s: 4, 60 s + over: 5)			0.0237	0.003
Visited Japan (yes, no)			0.2176	0.001
Got any health information			0.2101	0.001
Control: *Mari Info Japan* (*n* = 531)	0.1434	0.1286		
Gender			0.2149	0.015
Age (18-20 s: 1, 30 s: 2, 40 s: 3, 50 s: 4, 60 s + over: 5)			0.8086	0.069
Visited Japan (yes, no)			0.2306	0.001
Got any health information			0.2353	0.001

^a^*CSQ-8* 8-item Client Satisfaction Questionnaire, ^b^*SE* Standard Error, Multiple Regression

**Fig. 2:**
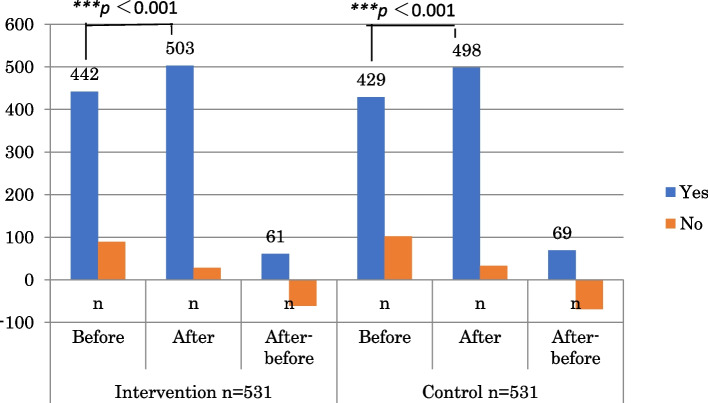
Motivation changed; Willingness to follow the health information compared with intervention and control groups

Figure 2 shows improvements in the participants’ motivation and willingness to follow Japanese health information after the interventions. The improvements in both the intervention and control groups were statistically significant (p < 0.001). However, the difference in changes in motivation between the two groups was not statistically significant by the chi-squared test (*p* = 0.258).

## Discussion

### Principal findings

In this study, both the intervention group and the control group showed improvements in health knowledge levels. The group that played the *Sa-Chan game* showed more satisfaction than the control group. Both groups indicated that they were willing to follow the health information they received to stay safe and healthy in Japan.

Both groups increased their knowledge of Japanese health care after the interventions. The high scores indicated that the participants learned more through a simple technique. We believe that the participants wanted to gain a deeper understanding of each topic. In the intervention group, the participants’ demographic backgrounds regarding gathering information before this study and the number of times they visited Japan were associated with understanding information topics. In the control group, the participants’ backgrounds regarding gathering information before this study were correlated with understanding information topics.

Both the intervention and control groups increased their CSQ-8 scores significantly after the interventions. Gathering Japanese health information is not easy for foreign visitors, whether they have already visited or are potential visitors. On the other hand, during our study, the participants’ health concerns were partly alleviated because they gained relevant knowledge through a visually pleasant method. They could access information that they did not know.

The intervention group had higher CSQ-8 scores than the control group. The game method was more satisfying than only watching a cartoon because it required thinking and providing answers to the situations and questions. The use of a game involves participation. In other words, the participants needed to think quickly and answer. This finding is similar to the results of a study in New York that used an interactive computer game for adults to increase their skills and self-efficacy for safer sex and HIV prevention (26). The researchers used a program structured as a time travel adventure game [28]. Our results were similar to those of a study on educating German medical students about earthquakes using a board game [29]. Another similar effect observed in a previous study was that the unsupervised usage of an educational game, "L'Affaire Birman", was able to improve insulin titration and carbohydrate quantification results for children with type 1 diabetes [30, 31].

Both the intervention and control groups showed an association of sex, age, number of visits to Japan, and previous Japanese health information received with CSQ-8 scores. Women 60–69 years old had a higher score than the other age groups. The reason for women’s higher satisfaction was that women were more concerned about health-related issues than men. A study in Germany showed that more women collected necessary health information on the internet than men [32]. The study explained that women have more experiences, experience greater enjoyment in health-related checks, and possess greater social willingness. Women care about their social responsibilities, suggesting that their needs should be considered when presenting health-related information on the internet [32].

Before this study, we thought that young people would have higher CSQ-8 scores than older age groups; however, the results showed the opposite, even though young adults use the internet in greater numbers. Medlock et al. found that regular internet users in the Netherlands were more likely to receive health information [33]. Senior citizens tend to be more concerned about health. This study found that those planning to visit Japan presented high satisfaction scores after both interventions. One explanation is that those without any prior information felt satisfied because they were given the information for the first time.

The participants wanted to follow the information to stay healthy in Japan. After the intervention, the participants acquired information about health and safety in Japan more readily. We used disease prevention information and willingness from the IMB model [26] for this study. The use of this model in this study was to increase the desire for preventive action by providing the necessary disease prevention, health promotion and care system information in Japan. The model has been successfully used to provide information for many disease domains in the United States related to mammography screening for African American women. A study found that the individual relevancy of information, procedural and systematic behavioral skills, and motivation improved adherence to breast cancer screening [27].

It is possible that the responses were influenced by the fact that this study was conducted during the COVID-19 pandemic. During the three years of the pandemic, the number of overseas visitors to Japan was greatly reduced, and participants stayed in their home countries. Because they had no plans to travel abroad in the near future, they had fewer concerns or anxiety about overseas travel when they participated in this study.

### Strengths and weaknesses

The main strength of this study is in determining an effective and interesting method of conveying Japanese health information to international visitors of any age group. Our study provided pertinent health information using current technology through a digital animation and quiz instead of a common guidebook or brochure. This method suits current societal trends and is more enjoyable for participants worldwide. It is not easy for international visitors to attain information to navigate health systems in other countries where their mother tongue is not commonly used. Moreover, few studies have assessed the knowledge level of health information, behavioral changes, or satisfaction in a developed country outside Europe or North America.

A limitation of this study is that only participants who registered with detailed demographic information could participate in this study. In addition, the survey was conducted among participants in three major Western countries, even though the majority of visitors to Japan have mainly come from East Asia in the last few years. This could have resulted in biased opinions. Therefore, generalizing the results of this study to other countries is difficult. It is difficult at this time to make comparisons with previous studies because very few studies have tested the effectiveness of similar interventions. Therefore, studies on similar interventions in other countries (regions) are needed.

Since the results of our analyses showed that there was little impact of participants’ previous visits to Japan on their outcome scores, we considered the bias to be small and conducted the analyses together in this study.

### Implications and future research

Like many other countries, Japan is promoting tourism as a source of revenue from economic activity; hence, it is vital for visitors to stay healthy. It is important to reflect on the results of this study in policy-making and incorporate them into campaigns when promoting international travel. As this study was conducted online, some participants did not actually visit Japan. Future research needs to investigate whether the content and methods of this information can be evaluated against actual visits.

## Conclusion

This study indicated the potential use of a distinctive education strategy through a unique game for overseas visitors to stay healthy and safe in Japan. Both the intervention and control groups had greater levels of knowledge and CSQ-8 scores after than before the interventions. The participants showed slightly higher satisfaction with the *Sa-Chan game* intervention than with the animation intervention. Both the intervention and control groups were willing to follow the health information. We conclude that host nations have obligations to provide useful and practical information in an attractive and effective way to welcome overseas visitors.

## Supplementary Information

**Additional file 1.** 

## Data Availability

Please contact the first author for raw data.

## Abbreviations

CSQ-8: 8-Item Client Satisfaction Questionnaire
COVID-19: Coronavirus disease 2019
IMB: Information-motivation-behavioral skills model
RCT: Randomized controlled trial
UMIN-CTR: University Hospital Medical Information Network Center Clinical Trials Registry
