# Supplementary material for: Japanese health and safety information for overseas visitors: a randomized controlled trial
Source: BMC Public Health. 2023 Jun 21;23:1194. doi: 10.1186/s12889-023-16117-5 (PMC10283307; doi:10.1186/s12889-023-16117-5)
Supplement: Supplementary file 1 — Additional file 1. [file 12889_2023_16117_MOESM1_ESM.docx]

Survey 1: Before intervention

Dear Participant,

We are interested in your honest opinion about health information for visitors to Japan. Please respond to these questions based on your current knowledge. We also welcome your comments and suggestions. This survey for academic research is anonymous, so no one will know what answers you provide. When you submit your responses, you consent to be a participant for our research. This short survey takes about 10 minutes to complete. Thank you for your time and help with this study.

Sincerely,

Prof. Mariko Nishikawa

The University of Human Environments, Japan

**Questioner for participants**:

**Section Ⅰ.** Socio-demographic characteristics

1. Gender: 🞏 Male 🞏 Female
2. Age: 🞏 18-19 🞏 20-29 🞏 30-39 🞏 40-49

🞏 50-59 🞏 60-69 🞏 Over 70

1. Your level of education:

🞏 University graduate 🞏 Junior college 🞏 High school or less

1. Have you ever visited Japan?  🞏 Yes 🞏 No
2. If, Yes. How many times have you visited Japan?

🞏 Once 🞏 More than once: _____times

1. Do you plan to visit Japan when coronavirus goes away? 🞏 Yes 🞏 No
2. Have you ever gotten any information about Japanese health or/and safety?

🞏 Yes 🞏 No

1. If, Yes. Where or How? (Check any that apply)

🞏 Health facility 🞏 Internet 🞏 Brochure 🞏 Travel guidebook 🞏 other ( )

**Section Ⅱ.**

Please choose your optimal level of knowledge about health information in Japan.

1. How would you rate the quality of information you received?

| 4 *Excellent* | 3 *Good* | 2 *Fair* | 1 *Poor* |
| --- | --- | --- | --- |

1. Did you get the kind of information you wanted?

| 1 *No, definitely not* | 2 *No, not really* | 3 *Yes, Generally* | 4 *Yes, Definitely* |
| --- | --- | --- | --- |

1. To what extent has our information met your needs?

| 4 *Almost all of my needs have been met* | 3 *Most of my needs have been met* | 2 *Only a few of my needs have been met* | 1 *None of my needs have been met* |
| --- | --- | --- | --- |

1. If a friend were in need of similar help, would you recommend our information to him or her?

| 1 *No, definitely not* | 2 *No, I don’t think so* | 3 *Yes, I think so* | 4 *Yes, definitely* |
| --- | --- | --- | --- |

1. How satisfied are you with the amount of help you received?

| 1 *Quite dissatisfied* | 2 *Indifferent or mildly dissatisfied* | 3 *Mostly satisfied* | 4 *Very satisfied* |
| --- | --- | --- | --- |

1. Has the information you received helped you to deal more effectively with your problems?

| 4 *Yes, they helped a great deal* | 3 *Yes, they helped somewhat* | 2 *No, they really didn’t help* | 1 *No, they seemed to make things worse* |
| --- | --- | --- | --- |

1. In an overall, general sense, how satisfied are you with the information you received?

| 4 *Very satisfied* | 3 *Mostly satisfied* | 2 *Indifferent or mildly dissatisfied* | 1 *Quite dissatisfied* |
| --- | --- | --- | --- |

1. If you were to seek help again, would you come back to our information?

| 1 *No, definitely not* | 2 *No, I don’t think so* | 3 *Yes, I think so* | 4 *Yes, definitely* |
| --- | --- | --- | --- |

Are you likely to follow this information yourself?

| 4 *Already follow* | 3 *Yes, I am going to* | 2 *May be not* | 1 *No definitely not* |
| --- | --- | --- | --- |

Do you understand how to deal with the following topics in Japan?

Pleases click ☑.

| No | Items | | **Yes** | **No** |
| --- | --- | --- | --- | --- |
| 1 | | *Health Promotion and safety* | □ | □ |
| 2 | | *Pay Medical Expenses* | □ | □ |
| 3 | | *Medical System* | □ | □ |
| 4 | | *Language Communication* | □ | □ |
| 5 | | *Informed Consent* | □ | □ |
| 6 | | *Choose a Hospital* | □ | □ |
| 7 | | *Pick up an Infection* | □ | □ |
| 8 | | *Quality of Nursing Care* | □ | □ |
| 9 | | *Quality of the Medicine* | □ | □ |
| 10 | | *Lifestyle Difference* | □ | □ |
| 11 | | *Eye Contact* | □ | □ |
| 12 | | *Protection of Privacy* | □ | □ |
| 13 | | *Directions in a Hospital* | □ | □ |
| 14 | | *Dealing with Medical Staff* | □ | □ |
| 15 | | *Emergency Care* | □ | □ |

Intervention (*Sa-chan game* or *Mari Info Japan*): four-minute duration.

After the intervention is complete, please answer the next section.

Survey 2: After intervention

**Section Ⅰ.**

Please answer based on the animation you just saw about health information in Japan.

1. How would you rate the quality of information you received?

| 4 *Excellent* | 3 *Good* | 2 *Fair* | 1 *Poor* |
| --- | --- | --- | --- |

1. Did you get the kind of information you wanted?

| 1 *No, definitely not* | 2 *No, not really* | 3 *Yes, Generally* | 4 *Yes, Definitely* |
| --- | --- | --- | --- |

1. To what extent has our information met your needs?

| 4 *Almost all of my needs have been met* | 3 *Most of my needs have been met* | 2 *Only a few of my needs have been met* | 1 *None of my needs have been met* |
| --- | --- | --- | --- |

1. If a friend were in need of similar help, would you recommend our information to him or her?

| 1 *No, definitely not* | 2 *No, I don’t think so* | 3 *Yes, I think so* | 4 *Yes, definitely* |
| --- | --- | --- | --- |

1. How satisfied are you with the amount of help you received?

| 1 *Quite dissatisfied* | 2 *Indifferent or mildly dissatisfied* | 3 *Mostly satisfied* | 4 *Very satisfied* |
| --- | --- | --- | --- |

1. Has the information you received helped you to deal more effectively with your problems?

| 4 *Yes, they helped a great deal* | 3 *Yes, they helped somewhat* | 2 *No, they really didn’t help* | 1 *No, they seemed to make things worse* |
| --- | --- | --- | --- |

1. In an overall, general sense, how satisfied are you with the information you received?

| 4 *Very satisfied* | 3 *Mostly satisfied* | 2 *Indifferent or mildly dissatisfied* | 1 *Quite dissatisfied* |
| --- | --- | --- | --- |

1. If you were to seek help again, would you come back to our information?

| 1 *No, definitely not* | 2 *No, I don’t think so* | 3 *Yes, I think so* | 4 *Yes, definitely* |
| --- | --- | --- | --- |

Are you likely to follow this information yourself?

| 4 *Already follow* | 3 *Yes, I am going to* | 2 *May be not* | 1 *No definitely not* |
| --- | --- | --- | --- |

Any comments or suggestions?

______________________________________________________________________

Do you understand how to deal with the following topics in Japan?

Pleases click ☑.

| No | Items | | **Yes** | **No** |
| --- | --- | --- | --- | --- |
| 1 | | *Health Promotion and safety* | □ | □ |
| 2 | | *Pay Medical Expenses* | □ | □ |
| 3 | | *Medical System* | □ | □ |
| 4 | | *Language Communication* | □ | □ |
| 5 | | *Informed Consent* | □ | □ |
| 6 | | *Choose a Hospital* | □ | □ |
| 7 | | *Pick up an Infection* | □ | □ |
| 8 | | *Quality of Nursing Care* | □ | □ |
| 9 | | *Quality of the Medicine* | □ | □ |
| 10 | | *Lifestyle Difference* | □ | □ |
| 11 | | *Eye Contact* | □ | □ |
| 12 | | *Protection of Privacy* | □ | □ |
| 13 | | *Directions in a Hospital* | □ | □ |
| 14 | | *Dealing with Medical Staff* | □ | □ |
| 15 | | *Emergency Care* | □ | □ |

Please provide any opinions about health information to visit Japan.

Thank you! (ありがとうございます)
